# Field‐in‐field breast planning for a jawless, double‐stack MLC LINAC using flattening‐filter‐free beams

**DOI:** 10.1002/acm2.12722

**Published:** 2019-10-16

**Authors:** Robert Morris, Eric Laugeman, Jessica Hilliard, Imran Zoberi, Ana Heerman, Geoffrey Hugo, Sasa Mutic, Bin Cai

**Affiliations:** ^1^ Department of Radiation Oncology Washington University St. Louis MO 63110 USA

**Keywords:** 6FFF, field‐in‐field radiotherapy planning, halcyon treatment planning

## Abstract

**Background:**

This study intends to develop an efficient field‐in‐field (FiF) planning technique with the Eclipse treatment planning system (TPS) to determine the feasibility of using the Halcyon treatment delivery system for 3D treatment of breast cancer.

**Methods:**

Ten treatment plans were prepared on the Halcyon treatment planning system and compared to the same patients’ clinically delivered TrueBeam plans which used flattened 6 MV and 10 MV beams. Patients selected for this study were treated via simple, tangential breast irradiation and did not receive radiotherapy of the supraclavicular or internal mammary lymph nodes. Planning target volumes (PTV) volumes ranged from 519 cc to 1211 cc with a mean target volume of 877 cc. Several planning techniques involving collimator, gantry rotation, and number of FiF segments were investigated as well as the use of the dynamically flattened beam (DFB) — a predefined MLC pattern that is designed to provide a flattened beam profile at 10 cm depth on a standard water phantom. For comparison, the clinically delivered TrueBeam plans remained unaltered except for normalization of the target coverage to more readily compare the two treatment delivery techniques.

**Results:**

Using the physician defined PTV, normalized such that 98% of the volume was covered by 95% of the prescribed dose, the Halcyon plans were deemed clinically acceptable and comparable to the TrueBeam plans by the radiation oncologist. Resulting average global maximum doses in the test patients were identical between the TrueBeam and Halcyon plans (108% of Rx) and a mean PTV dose of 102.5% vs 101.6%, respectively.

**Conclusions:**

From this study a practical and efficient planning method for delivering 3D conformal breast radiotherapy using the Halcyon linear accelerator has been developed. When normalized to the clinically desired coverage, hot spots were maintained to acceptable levels and overall plan quality was comparable to plans delivered on conventional C‐arm LINACs.

## 1 INTRODUCTION

Breast carcinoma is the most commonly diagnosed cancer in females. About one in eight women in the United States (12.4%) will develop invasive breast cancer over the course of their lifetime.^1^ Radiotherapy, in conjunction with breast conservation surgery and chemotherapy, plays a major role in the treatment of these cancers.^2^ Breast radiotherapy is typically delivered by conventional 3D beams or intensity modulated radiation therapy (IMRT). While IMRT is often better suited to treat complex targets with involved lymph nodes,^3^ conventional 3D tangent beams continue to make up a large percentage of radiotherapy treatments with acceptable clinical outcomes and widespread availability.

Recently, a compact ring‐shape medical linear accelerator (LINAC) system, Halcyon (Varian Medical Systems, Inc.), was released. The Halcyon system provides a single 6FFF beam with double‐stack multi‐leaf collimator (MLC) beam shaping system and faster gantry rotation when compared with conventional C‐arm LINACs. TrueBeam linear accelerators can deliver treatments at a maximum gantry rotation of one rotation per minute vs four rotations per minute with Halcyon. Maximum dose rate for a flattened 6 MV or 10 MV is 600 MU/min at isocenter for TrueBeam and 800 MU/min at isocenter for the 6FFF beam provided on the Halcyon LINAC. Halcyon was designed to address the global need for access to radiation therapy with integrated imaging guidance, improved clinical efficiency, and shorter installation and commissioning time as well as operational demands.^4^, ^5^, ^6^ However, the jawless design and unflattened beam provide some challenges when creating breast 3D plans with the FiF techniques. The jawless design might suggest that there is increased out‐of‐field leakage dose to the patient compared to traditional C‐arm accelerators where jaws are needed to minimize this out‐of‐field leakage. For example, Varian TrueBeam jaws collimate the radiation that is not defined by the MLC aperture and minimize interleaf leakage (radiation that permeates two adjacent leaves of the same bank). There are two sets, one for each x‐y collimation direction. They are 78 mm thick along the beam axis and are comprised of a tungsten alloy. Halcyon uses a double‐stacked MLC arrangement with thicker leaves to account for a lack of jaws. The Millenium‐120 MLCs fitted to most TrueBeams have 67‐mm‐thick tungsten leaves, whereas the Halcyon has 77 mm thick leaves in each bank, creating a combined thickness of 154 mm which results in an average transmission factor of 0.0047 vs 0.015 for a Millenium‐120 system. Because the MLCs are offset in the direction perpendicular to leaf travel, the interleaf leakage is attenuated by the distal leaf bank. This design with thicker, offset leaves obviates the need for jaws.

Typically, the use of 6 or 10 MV flattened beam and a set of static MLC apertures can provide dose uniformity that is clinically acceptable. To better control the dose homogeneity, the field‐in‐field (FiF) technique is also often used in breast 3D planning.^7^, ^8^, ^9^ This method uses multiple, planner‐defined MLC segments that reduce hot spots and improve dose homogeneity. Traditional flattened beams typically have flatness values under 3% for an isocentrically placed phantom at 10cm depth. The 6FFF beams used by Halcyon have flatness values of 10%–20% under the same setup conditions. Using the conventional tangent beam arrangement on a breast target would result in clinically unacceptable dose heterogeneity, usually with large hot spots in excess of 135% of the prescription dose. If the conventional FiF planning technique is directly applied, it typically requires a large number of static MLC segments to control the hot and cold spots, increasing the complexity of the plan and required planning time. In this study, we develop and evaluate a planning strategy for the Halcyon accelerator to produce clinically acceptable breast FiF plans using 6FFF beams. Differences in target coverage and organ at risk (OAR) doses as a result of the 6FFF beam quality were examined as well as patient‐specific quality assurance (PSQA) delivery and analysis methods.

## 2 METHODS

### 2.1 Radiotherapy delivery equipment

**Figure 1:**
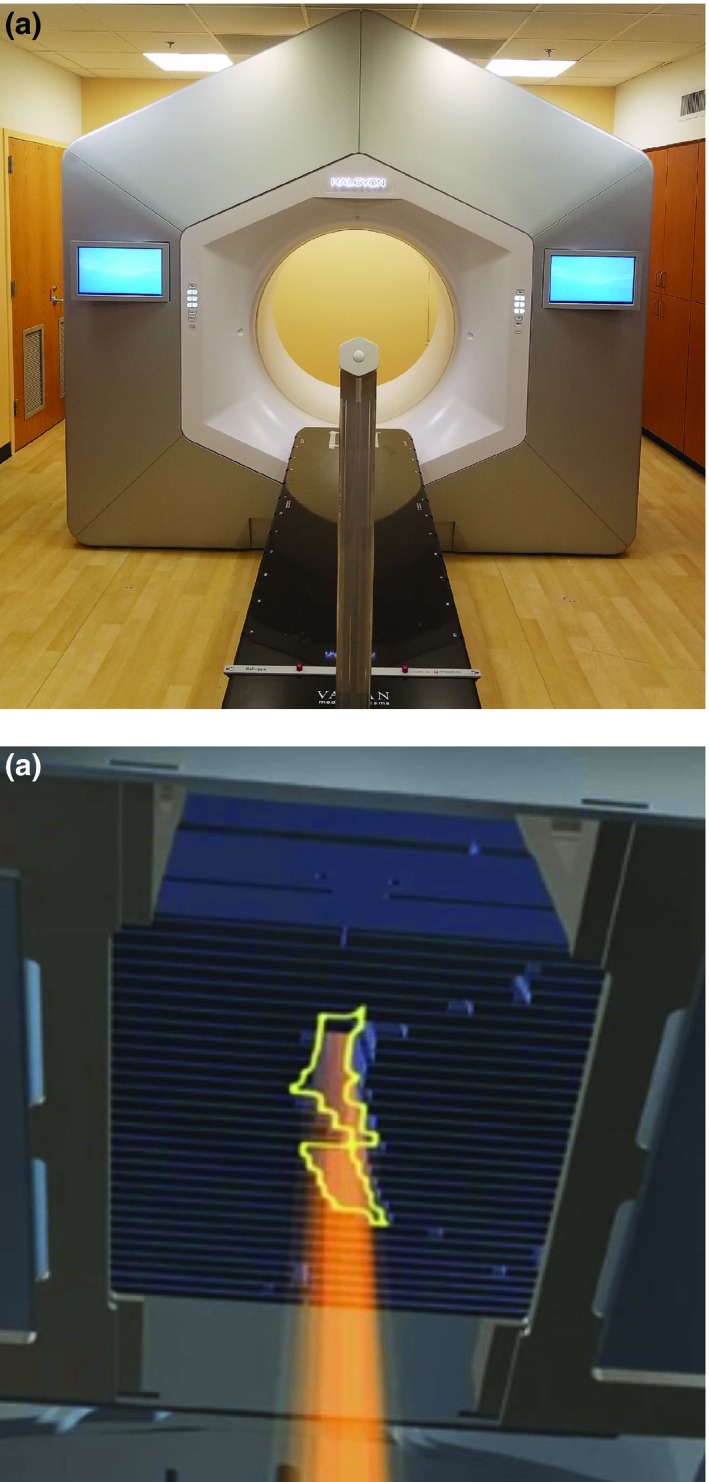
(a) Halcyon linear accelerator at Washington University in St Louis. (b) Detectors‐eye‐view of the lower MLC leaf bank. MLC, multi‐leaf collimator.

As shown in Fig. 1(a), Halcyon is a ring gantry LINAC with 100 cm diameter bore size and is capable of providing a 6FFF beam utilizing a compact gantry head design. This compact head allows a 1 m diameter patient bore LINAC to be installed in the same room as required for a conventional C‐arm LINAC. A typical C‐arm LINAC has an effective “bore diameter” of approximately 80 cm, since the physical measurement from the treatment head enclosure to mechanical isocenter is 40 cm. Halcyon employs a staggered, double‐stack MLC system, as illustrated in Fig. 1(b), which is the only custom beam shaping device between the x‐ray source and the patient. The MLC leaf width is 1.0 cm when projected at isocenter with 100 cm source‐axis distance (SAD). The proximal and distal layers are offset, or staggered, by 50% of the leaf width such that the effective resolution of the leaf stack pair becomes 0.5 cm at isocenter. The maximum leaf speed is 5.0 cm/s at isocenter with 100% interdigitation capability. There are 29 leaf‐pairs leafs in the proximal layer and 28 leaf‐pairs in the distal layer with the maximum field size of 28 × 28 cm^2^ at isocenter. One of the advantages of the double‐stack design is that the combined transmission is less than 0.01%, negating the need for physical jaws to reduce peripheral dose to nontargeted organs.

### 2.2 Patient selection

Ten breast patients clinically treated with 3D FiF technique on C‐arm LINACs (TrueBeam, Varian Medical System) from our institution were randomly selected and anonymized. To confine the study to feasibility of utilizing Halcyon for tangential breast radiotherapy, patients with involved supraclavicular, axillary, or internal mammary lymph node involvement were excluded. Across the 10 patients selected, two physicians and three dosimetrists were involved in the planning and approval of the treatment plans. This particular selection was performed to minimize potential bias due to a single planner. The prescribed dose to the PTV was either 4256cGy (266cGy × 16 fractions) or 5040cGy (180cGy × 28 fractions). Clinically, 6 MV beams were utilized for eight of the 10 patients with 10 MV for the remaining two due to increased breast bridge separation (the distance between the medial and lateral borders of the breast tissue at the middle of the breast). No couch kicks were utilized in the clinical plans for any of the patients selected. Planning target volumes (PTV) ranged from 519 cc to 1211 cc with a mean of 877.4 cc. Due to the intrinsic differences in MLC and beam quality, the Halcyon patients were planned *de novo* but still using the same gantry angles as the TrueBeam plans. Collimator rotation and MLC shaped segments as well as respective field weighting differed from the TrueBeam plans in order to produce homogeneous plans with the FFF beam quality in as few steps as possible. For dosimetric comparison with the Halcyon FiF plans, the plan normalization was the only parameter modified on the clinically delivered TrueBeam plans. Each plan was normalized such that 98% of the PTV received at least 95% of the prescribed dose (V_95%Rx_ = 98%).

### 2.3 Halcyon FiF plan generation

The Halcyon FiF plans were generated in Eclipse 15.6 treatment planning system (Varian Medical Systems) with a standardized planning process specific to our clinic. The overall goal is to create as few FiF segments in an attempt to balance planning time (<30 min per patient) and complexity with clinically acceptable target coverage (V_95_> 98%), and to minimize the global maximum dose (<115%) relative to the prescribed dose. The following sections 2.3.1‐2.3.5 highlight the key steps and strategies during the planning process

#### 2.3.1 Field setup

**Figure 2:**
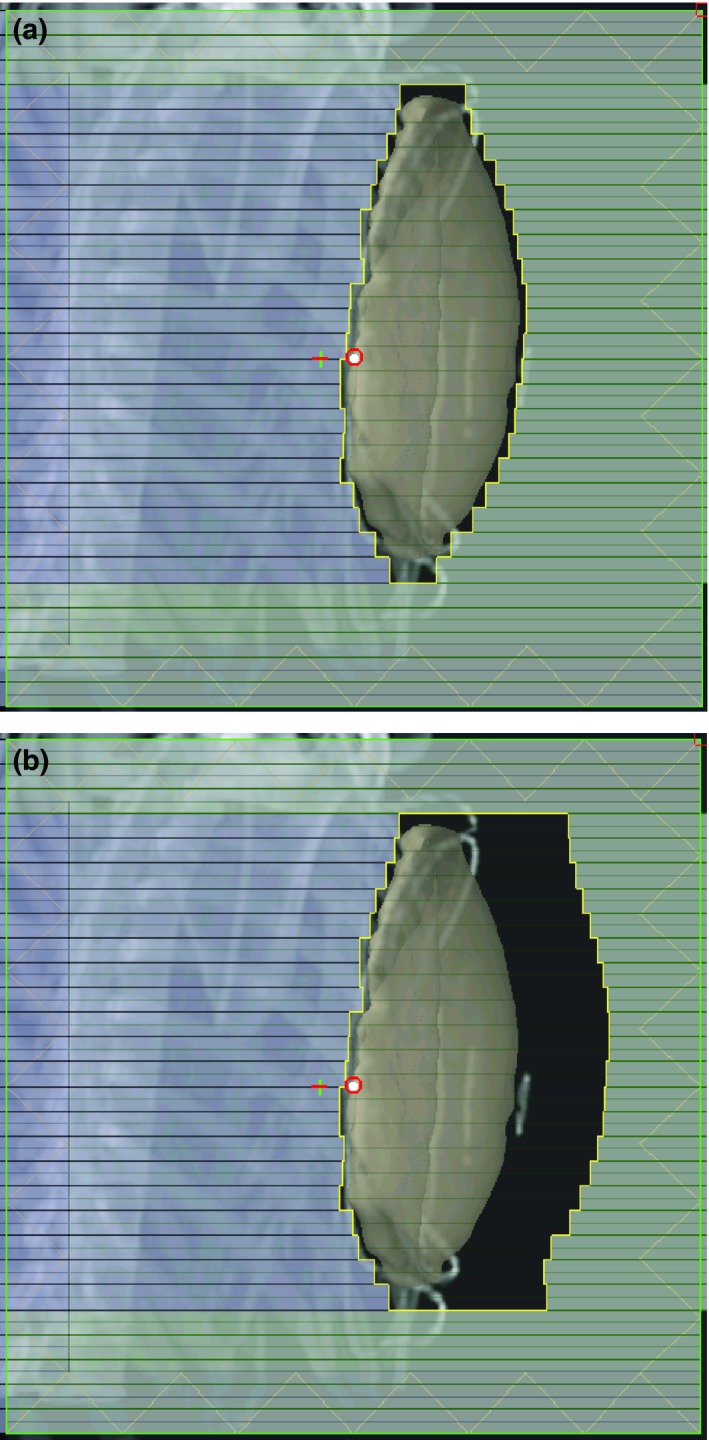
(a) BEV of isotropic 0.3 cm margin, circular MLC fit to PTV. (b) Flash added to anterior MLCs. BEV, beams‐eye‐view; MLC, multi‐leaf collimator; PTV, Planning target volumes.

Halcyon planning workflow starts with the clinically delivered, physician defined gantry angles and CT simulation‐defined isocenter (mid‐bridge). The MLCs are fit to the beams‐eye‐view (BEV) contour of the PTV with a 0.3 cm isotropic margin. At this point the BEV is evaluated with the heart and PTV volume visualization turned on. Typically in clinical plan, the physician will define the medial block edge by placing a block margin around the medial aspect of the PTV with consideration for minimizing heart dose. The block was designed based on individual patient’s geometry. In this study, we attempt to develop a simplified and standardized procedure to test the feasibility of FiF technique on Halcyon. Therefore, the heart block was not individually drawn for each patient. Also, at our clinic, this physician defined block edge is not modified by the planner, only MLCs from the other bank are moved to accomplish the FiF modulation. In this study, we attempt to develop a simplified and standardized procedure to test the feasibility of FiF technique on Halcyon. This is not the case in this study and MLCs from either bank were used to modulate dose as needed to achieve target coverage and minimize OAR doses. Then approximately 2 cm of flash is added on the nipple side of the target to account for potential respiratory motion and setup uncertainty (Fig. 2). Dose is then computed without normalization. Initial hotspots may exceed 140% or be as low as 95% depending on the patient anatomy. The subsequent planning steps focus on minimizing dose heterogeneity within the beams eve‐view.

#### 2.3.2 Dynamically flattened beam sequence

The initial tangent fields incorporate the use of the TPS dynamically flattened beam (DFB) option.^10^ The DFB is a predefined MLC sequence unique to Halcyon which sweeps the MLCs, whereas the beam is delivering dose to provide a flattened beam profile for a fixed gantry beam delivery. This profile, however, is only flattened at depth when delivered on a flat, homogeneous phantom. The DFB sequence does not take into account patient anatomical variations. The DFB tangents alone did not produce clinically acceptable dose homogeneity for any of our tested patients (global Dmax < 115% of prescription dose when the PTV was normalized such that V_98_ = 95%). However, we found that the use of DFB tangents as opposed to non‐DFB tangents markedly reduces the planning time by reducing the number of manually defined FiF segments that would be required to achieve a similar, clinically acceptable dose distribution.

**Figure 3:**
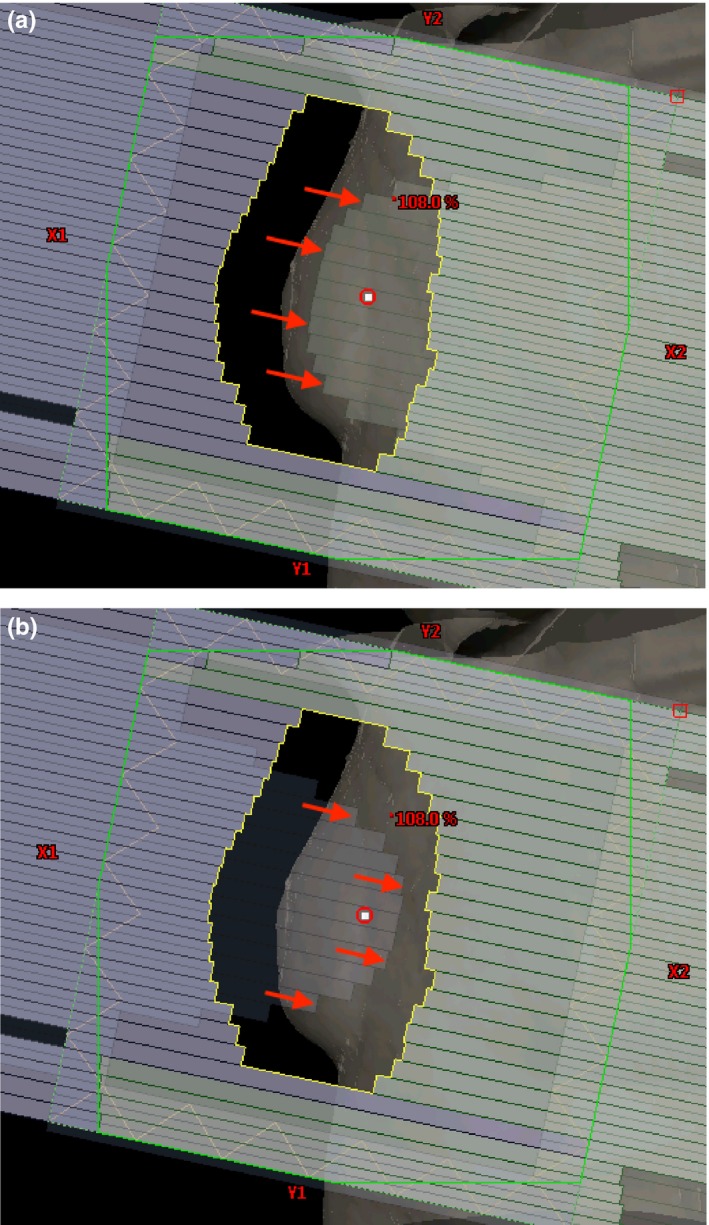
BEV of DFB sequence: (a) Proximal bank B (X2) opening pattern; (b) Proximal bank A (X1) closing pattern. BEV, beams‐eye‐view.

The DFB sequence (Fig. 3) is delivered by starting beam delivery with the field fully blocked by proximal leaf bank B (X2). During continuous beam‐on, these closed leaves open in a predefined sequence and park at the opposite field edge. Then the leaves of bank A (X1) initiate their closing sequence until the beam is again fully blocked. The DFB sequence is a binary option in the treatment planning system and has no change in control points as a function of patient anatomy, and only varies with field size. The number of monitor units for a DFB field is significantly increased compared to an open field that would deliver the same dose at treatment depth. This is because the beam is continuously being delivered while the MLCs sweep across so the high‐intensity portion of the beam is blocked to make a flat beam. Table 3 in the Results section outlines the overall MU difference in plans using the DFB vs TrueBeam. Details about how the leaf sequence is programmed to produce a flat beam are outside of the scope of this paper. Some details of DFB field are provided by Constantin et al [10].

#### 2.3.3 Collimator rotation optimization

**Figure 4:**
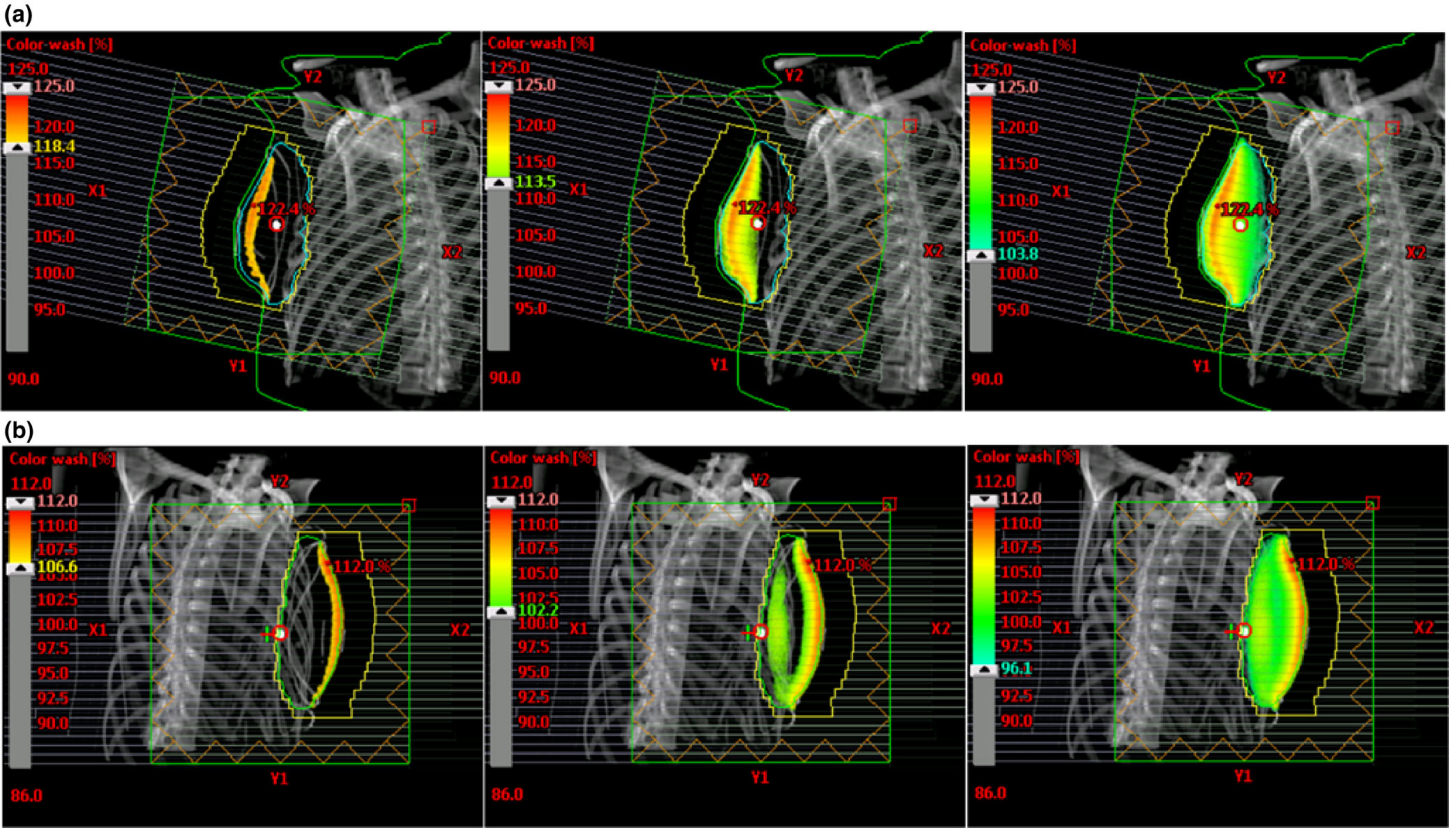
(a) Top row; Planning case in which the optimal collimator rotation was approx. 7 degrees. (b) Bottom row; Planning case in which collimator rotation was not required.

Optimal collimator rotation is needed to aid in subsequent modulation of hot spots with the highest resolution achievable with the MLC shaping system. A similar concept is used with TrueBeam, but is usually slightly different due to the changes in hot spot extent and location as a result of the FFF beam. The optimal rotation is determined after the initial dose calculation by analyzing the directions of isodose falloff. This refers to the general direction between two or more isodose lines. Practically, this is better determined through the use of dose color‐wash visualization in the BEV with the range selection slider bar. Mitigation of hot spots with regard to the movement limitations of the MLC is achieved by setting the collimator rotation angle such that the direction of leaf motion is parallel to the isodose falloff at the widest section of the breast in the medial‐lateral direction, typically at the level of the nipple (Fig. 4). Observed factors that influence optimal collimator rotation are breast volume, anatomic tissue distribution, and simulation setup conditions including slant board angle.

After optimal collimator angle is determined, the MLCs are re‐fitted to the PTV with the 0.3 cm isotropic margin and approximately 2 cm of flash added by manually selecting and dragging the leaves of the anterior banks for the two DFB tangent beams. Once dose is recomputed, the field weight of the DFB tangents is adjusted such that the global Dmax is minimized and/or evenly distributed in the craniocaudal axis.

#### 2.3.4 Manual FiF segments

**Figure 5:**
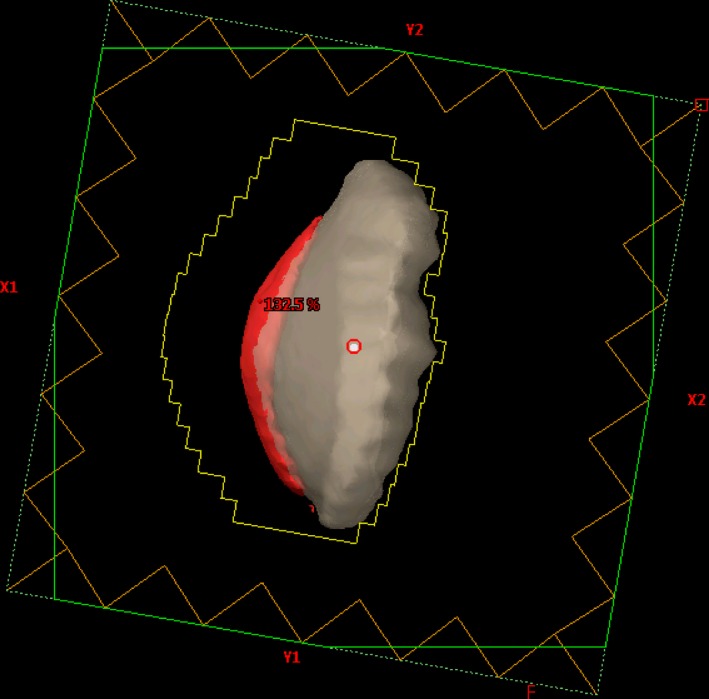
BEV of the initial field. An arbitrary isodose line relative to the maximum dose is chosen as a guide for placement of the MLC leaves such that approx. 1–2 cm of collective leaf motion results. BEV, beams‐eye‐view; MLC, multi‐leaf collimator.

The initial FiF segment typically utilized only the anterior leaf bank for hot spot reduction. For ease of MLC placement, an isodose is selected that yields a conventional FiF segment size in the BEV. In our clinic, the planning strategy is to select an isodose that is approximately 10%–15% lower than the hot spot but this technique does not work well for the 6FFF beam quality and typically results in a plan that has more FiF segments and required much more time to plan. An “acceptable” FiF segment with this planning strategy yields a 1–2 cm collective leaf motion from the previous segment (Fig. 5). This method attempts to limit the number of segments while keeping a dosimetrically meaningful number of monitor units (MU) per segment. Institutionally, we have a minimum restriction of 5 MU per segment for conventional LINAC FiF planning as well as Step‐and‐Shoot IMRT delivery. This reduces the uncertainty due to nonlinearities in the delivery of monitor units. The Halcyon FiF planning method has adopted this policy.

The process of selecting an isodose line as an MLC placement guide is repeated approximately 2–4 times for each tangent field at the initial gantry and collimator rotation angles resulting in 4–8 total FiF segments for the plan. These FiF segments do not utilize the DFB feature and are delivered in a step‐and‐shoot fashion with the LINAC in a beam‐hold state during MLC movements. Generally speaking, larger breast volumes will tend to require a higher number of FiF segments to control hot spots and improve low dose coverage.

#### 2.3.5 Field weighting

**Figure 6:**
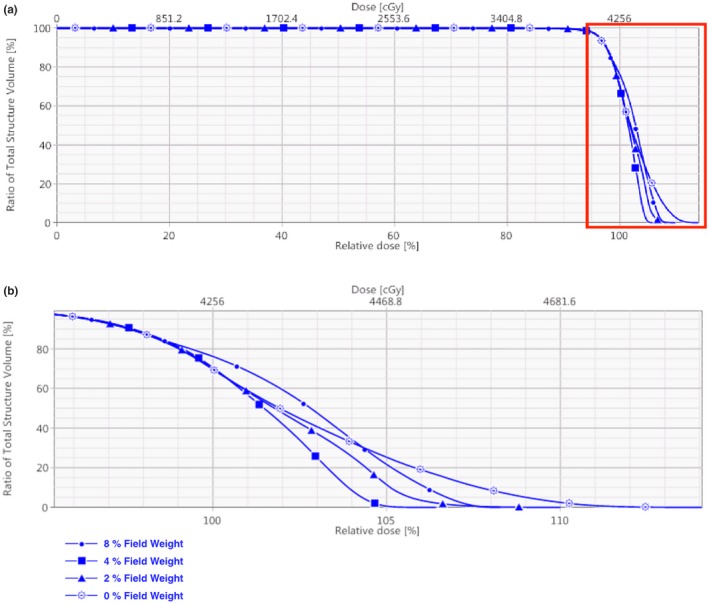
(a) Initial DVH of PTV for patient 05 using various field weights for the first 2 FiF segments. (b) Zoomed view of high dose region of DVH in Figure 6. For this segment, 4% field weight was optimal but optimal field weights will vary from patient to patient as well as segment to segment. DVH, dose volume histogram; PTV, Planning target volumes.

Field weighting is performed after each segment is added and dose recomputed. Weights are locked for all segments delivered at the same gantry angle such that the FiF segment being weighted trades monitor units with the initial DFB tangent beam of the same gantry angle. This method attempts to keep the global hot spots evenly distributed in the craniocaudal direction. Appropriate field weight for each segment is identified by actively monitoring the PTV coverage in the dose volume histogram (DVH) viewer. Initial FiF segments control the high‐dose slope of the DVH curve, whereas the final FiF segments control the low‐dose coverage of the target volume. Figure 6 demonstrates how the shape of the curve changes as a function of field weight for a single initial segment. The optimal field weight will vary based on MLC segment size, patient anatomy, and initial dose distribution. This strategy can be applied to all FiF planning and should be used to minimize the number for FiF segments needed and overall planning time.

In our testing, optimal field weight tends to straighten the DVH curve as much as possible while minimizing the high dose tail.

#### 2.3.6 Plan normalization

**Figure 7:**
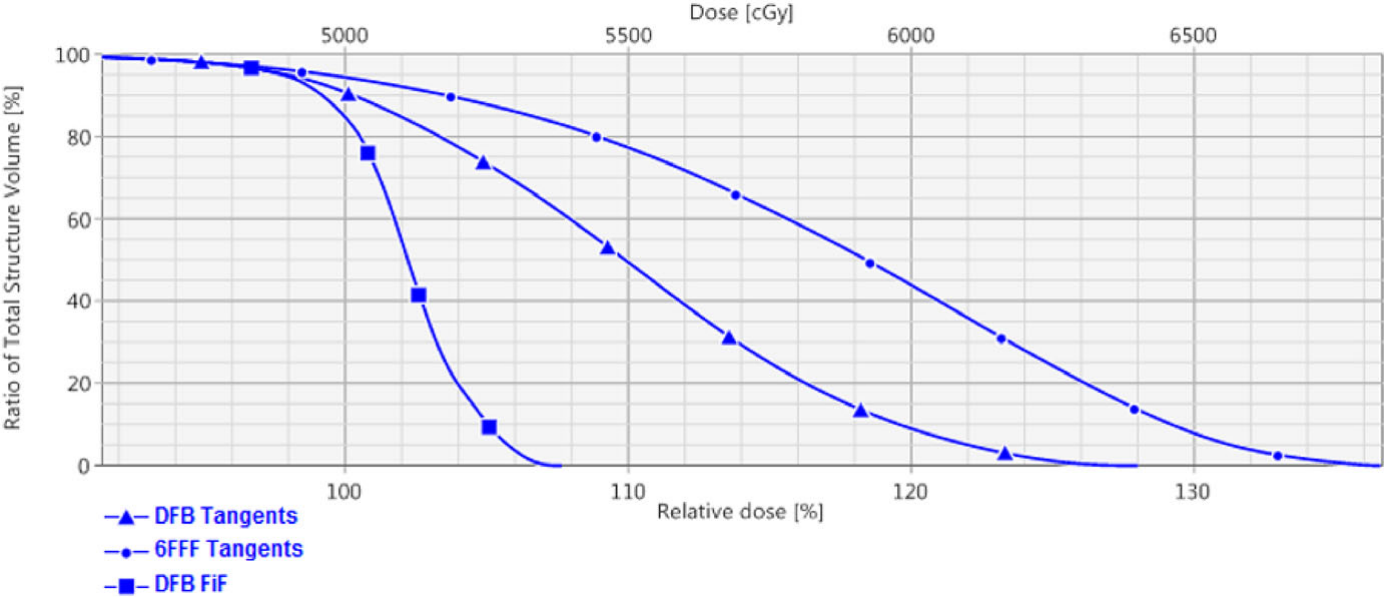
DVH comparison of PTV coverage using 6FFF tangent beams alone, the DFB tangents alone, and the DFB tangents paired with the manual FiF segments. All three plans were normalized such that 98% of the PTV volume was covered by 95% of prescription. DFB, dynamically flattened beam; DVH, dose volume histogram; PTV, Planning target volumes.

The final planning step involves normalization. In this planning study all patients were normalized such that 98% of the volume was covered by 95% of the prescribed dose. If the global D_max_ was in excess of 115% the normalization was reduced until the global D_max_ was below 115% of prescription. This situation was not encountered with any of the 10 test plans. In clinical practice the target coverage and normalization objectives are defined by physician preference. At our institution, it is standard practice to achieve a PTV coverage of V_95%_ = 98% (V_95_ = 95% minimum acceptable) with a global D_max_ less than 115% (Fig. 7)

### 2.4 Deliverability and plan measurement

In order to test the deliverability of the ten plans, PSQA was performed on each plan. First, ion chamber measurements were taken with a PTW 31010, in a solid water phantom (15 cm^3^). Passing criteria were percent dose deviation of less than ±3% from the expected dose. Second, portal dosimetry was performed for each field. A composite image was created, and gamma criteria was set according to the recommendations of AAPM task group 218 (3% dose difference, 2 mm distance‐to‐agreement, global normalization, 10% threshold with a tolerance level of 95% of pixels passing).^11^ Third, the plans were delivered to a Delta4 Phantom+ (ScandiDos, Sweden). The same criteria were set as for portal dosimetry on the composite dose delivered to the phantom.

## 3 RESULTS

### 3.1 Overall plan summary

All Halcyon plans were successfully generated within 30 min following the standardized process developed and were evaluated by a radiation oncologist that specializes in breast radiotherapy. Overall, each Halcyon plan tested was deemed clinically acceptable. The physician evaluated the organs at risk as well as target coverage via isodose and DVH analysis. Table 1 summarizes the key results for PTV dose across all ten patients. As mentioned before, for a fair comparison, all Halcyon and TrueBeam plans in this study are normalized such that 98% of the PTV volume is covered by 95% of the prescribed dose. The mean global D_max_ is the same for both the TrueBeam and Halcyon plans (108.0%). The standard deviation for the global D_max_ in the plans is 2.5% for TrueBeam and 2.6% for Halcyon. The mean PTV dose on the TrueBeam plans is hotter (102.5%) compared with the Halcyon plans (101.6%).

**Table 1:** Summary of Test Patient Results — PTV dose.

Patient	PTV volume	Site laterality	TB Global Dmax	H Global Dmax	TB mean PTV	H mean PTV
1	1069 cc	Right	106.7%	111.5%	101.1%	102.9%
2	709 cc	Left	108.8%	110.3%	103.3%	103.3%
3	519 cc	Left	109.1%	106.6%	103.6%	99.8%
4	909 cc	Right	106.4%	108%	103.1%	102%
5	687 cc	Right	105.9%	104.4%	100.5%	100.5%
6	566 cc	Left	113.2%	105.1%	107.3%	100.9%
7	1174 cc	Right	105.1%	105.7%	99.9%	99.8%
8	801 cc	Right	106.4%	107.2%	99.8%	101.5%
9	1129 cc	Left	107.6%	111%	103.1%	101.6%
10	1211 cc	Right	110.9%	110.3 %	103.7%	103.2%
Average	877.4 cc	40% Lt 60% Rt	108.0%	108.0%	102.5%	101.6%
Standard Deviation	257.4 cc	n/a	2.5%	2.6%	2.3%	1.3%

PTV, planning target volumes.

OAR doses were analyzed for the heart, ipsilateral lung, and contralateral lung. The mean dose for all TrueBeam plans was 97.96 cGy vs 149.58 cGy for the Halcyon plans. The average D_max_ of the heart for the left‐side target in Halcyon plans is 4450.1 cGy compared to 4403.4 cGy for TrueBeam Plans; the average D_max_ for the heart is 360.0 cGy for Halcyon plans with right‐side target compared to 474.9 cGy for TrueBeam Plans. Mean dose to the contralateral lung dose increased from 26.41 cGy to 39.0 cGy for the Halcyon plans. Ipsilateral mean lung dose decreased using the Halcyon plans from 676.3 cGy to 569.4 cGy. The mean OAR dose summary is listed in Table 2.

**Table 2:** Summary of Test Patient – mean OAR dose.

Patient	PTV Volume	TB mean Heart (cGy)	Hal mean Heart (cGy)	TB mean Ipsil. Lung (cGy)	Hal mean Ipsil. Lung (cGy)	TB mean Cont. Lung (cGy)	Hal mean Cont. Lung (cGy)
1	1069 cc	46.8	55.3	540.5	332.0	4.3	8.5
2	709 cc	225.6	353.2	574.6	583.1	170.2	187.3
3	519 cc	205.0	345.0	735.0	745.0	20.0	40.0
4	909 cc	45.0	70.0	610.0	460.0	5.0	15.0
5	687 cc	38.3	59.6	940.6	889.5	4.3	12.8
6	566 cc	149.0	280.9	919.3	872.5	8.5	25.5
7	1174 cc	46.8	63.8	740.5	655.4	34.0	46.8
8	801 cc	4.3	21.3	757.6	297.9	4.3	12.8
9	1129 cc	155.0	170.0	375.0	310.0	5.0	20.0
10	1211 cc	63.8	76.6	570.3	549.0	8.5	21.3
Average	877.4 cc	97.96	149.58	676.34	569.44	26.41	38.99
Std. Dev.	257.4 cc	78.27	129.01	175.84	221.73	51.44	78.27

TB, TrueBeam; Hal, Halcyon; PTV, planning target volumes.

The mean number of monitor units used for the Halcyon delivered plans was 732, whereas the TrueBeam delivered plans used 279. In Table 3, it is evident that for larger PTVs (particularly those that were clinically treated with 10 MV) more FiF segments are necessary with the Halcyon LINAC due to the shallower PDD_10_.

**Table 3:** Energy, MU, and Segments used.

Patient	PTV Volume	TB Energy	Hal Energy	TB FiF Segments	H FiF Segments	TB MU	Hal MU
1	1069 cc	6 MV	6FFF	5	7	302 MU	796 MU
2	709 cc	6 MV	6FFF	5	5	328 MU	736 MU
3	519 cc	6 MV	6FFF	4	6	244 MU	557 MU
4	909 cc	10 MV	6FFF	4	6	223 MU	574 MU
5	687 cc	6 MV	6FFF	3	4	300 MU	739 MU
6	566 cc	6 MV	6FFF	3	4	309 MU	713 MU
7	1174cc	6 MV	6FFF	6	6	306 MU	828 MU
8	801 cc	6 MV	6FFF	5	6	310 MU	844 MU
9	1129 cc	10 MV	6FFF	4	8	224 MU	677 MU
10	1211 cc	6 MV	6FFF	5	6	239 MU	857 MU

TB, TrueBeam; Hal, Halcyon; PTV, planning target volumes.

### 3.2 Sample plans

**Figure 8:**
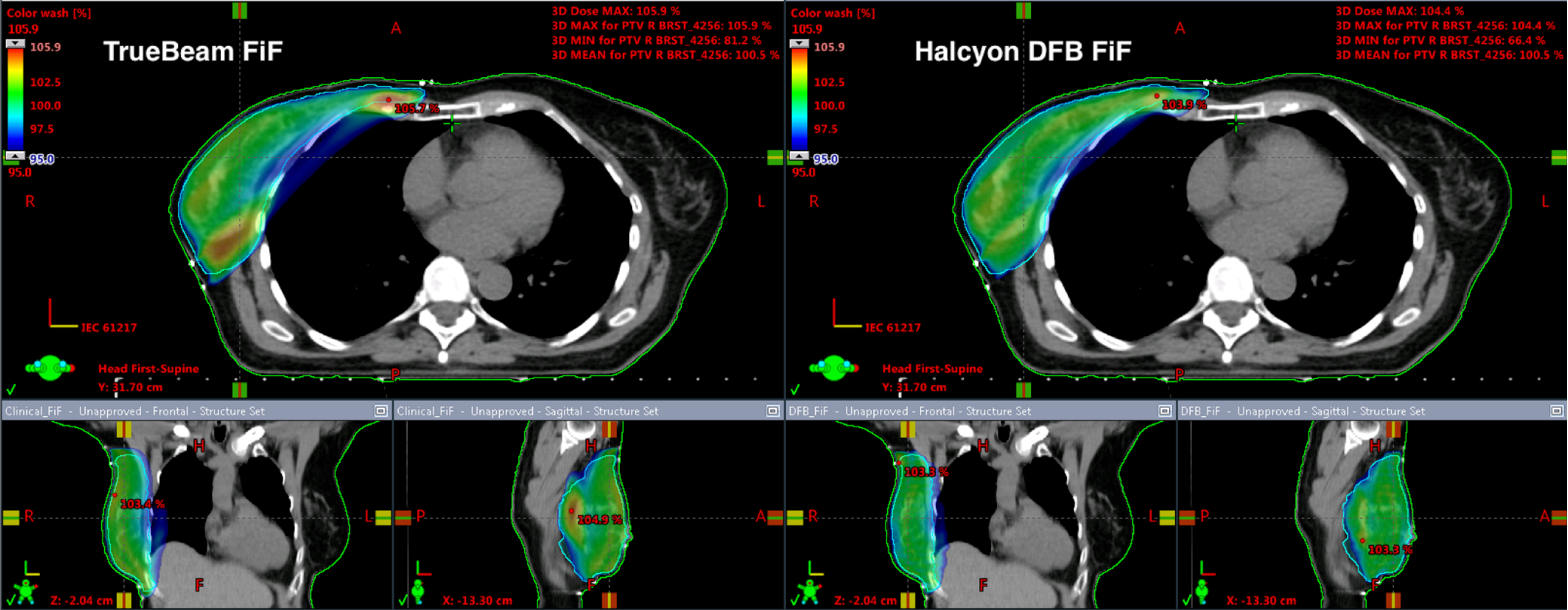
Qualitative analysis for patient 05: Bridge separation = 19 cm. PTV Volume = 687 cc. PTV, planning target volumes

**Figure 9:**
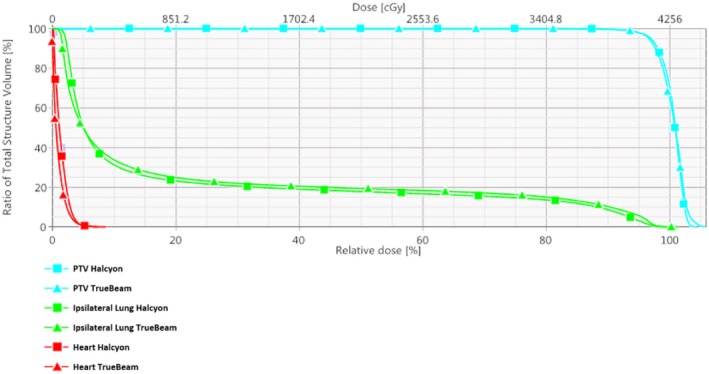
DVH comparison for patient 05: Bridge separation = 19 cm. PTV Volume = 687 cc. DVH, dose volume histogram; PTV, planning target volumes.

Figures 8 and 9 illustrate the dose distribution and DVH comparisons, respectively, of Halcyon and TrueBeam plans for test patient 05. This test plan is representative of a typical Halcyon plan in terms of resultant coverage and global Dmax. This patient had a bridge separation of 19 cm and a PTV volume of 687 cc which is within one standard deviation (257 cc) of the average PTV volume of the patients tested (877.4 cc). Qualitatively, the Halcyon plan has a more uniform dose in all three anatomical planes. The global D_max_ was 1.5% lower (105.9% vs 104.4%). Ipsilateral mean lung dose decreased from 375 cGy to 310 cGy, a 17.3% reduction. The DVH in Figure 9 shows comparable PTV coverage and OAR sparing between the two plans. Overall, the Halcyon plan only incorporated 1 more FiF segment compared to the TrueBeam plan (4 vs 3) (10).

**Figure 10:**
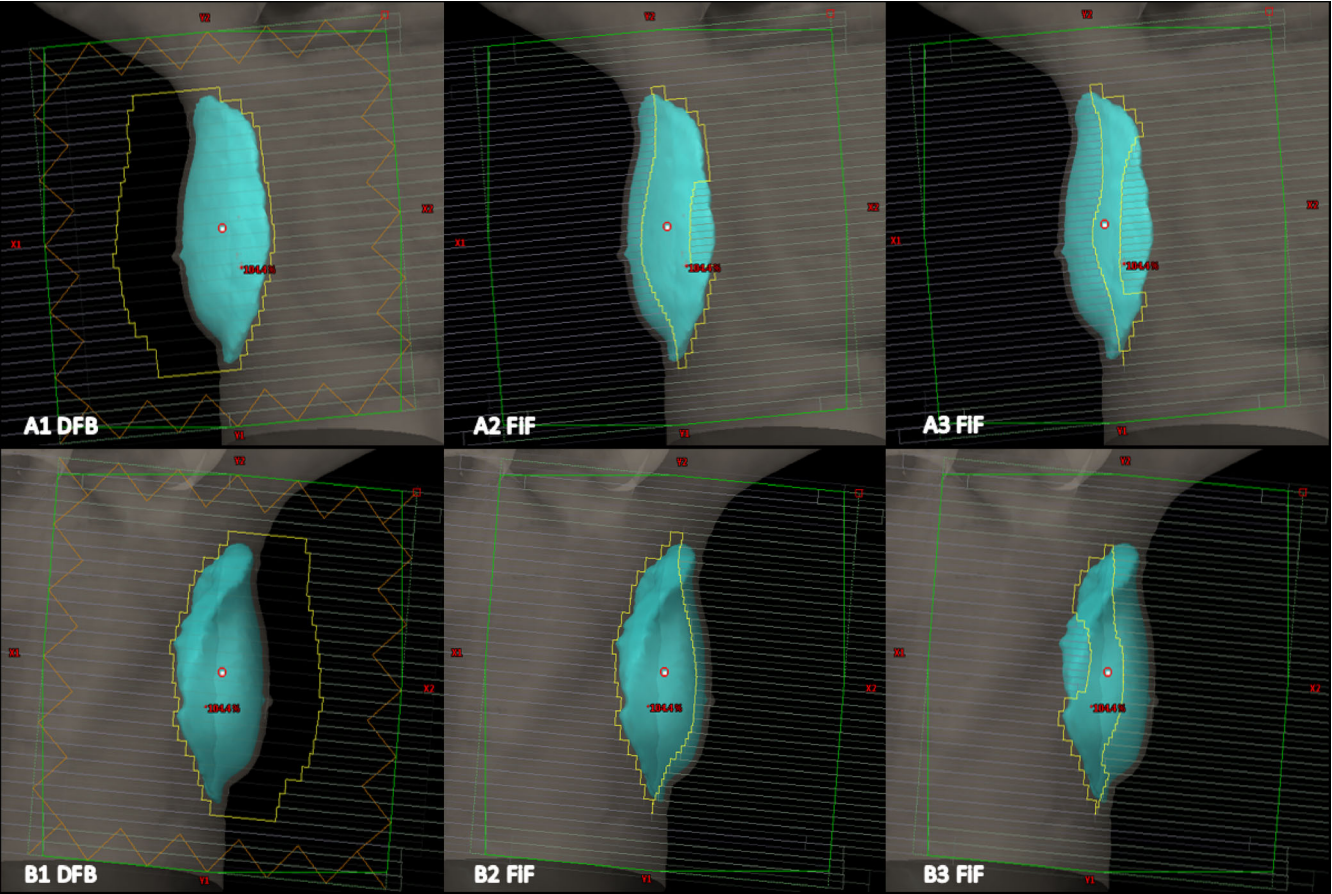
BEV of the DFB and FiF segments for patient 05: Bridge separation = 19 cm. PTV Volume = 687 cc. BEV, beams‐eye‐view; DFB, dynamically flattened beam; PTV, planning target volumes.

**Figure 11:**
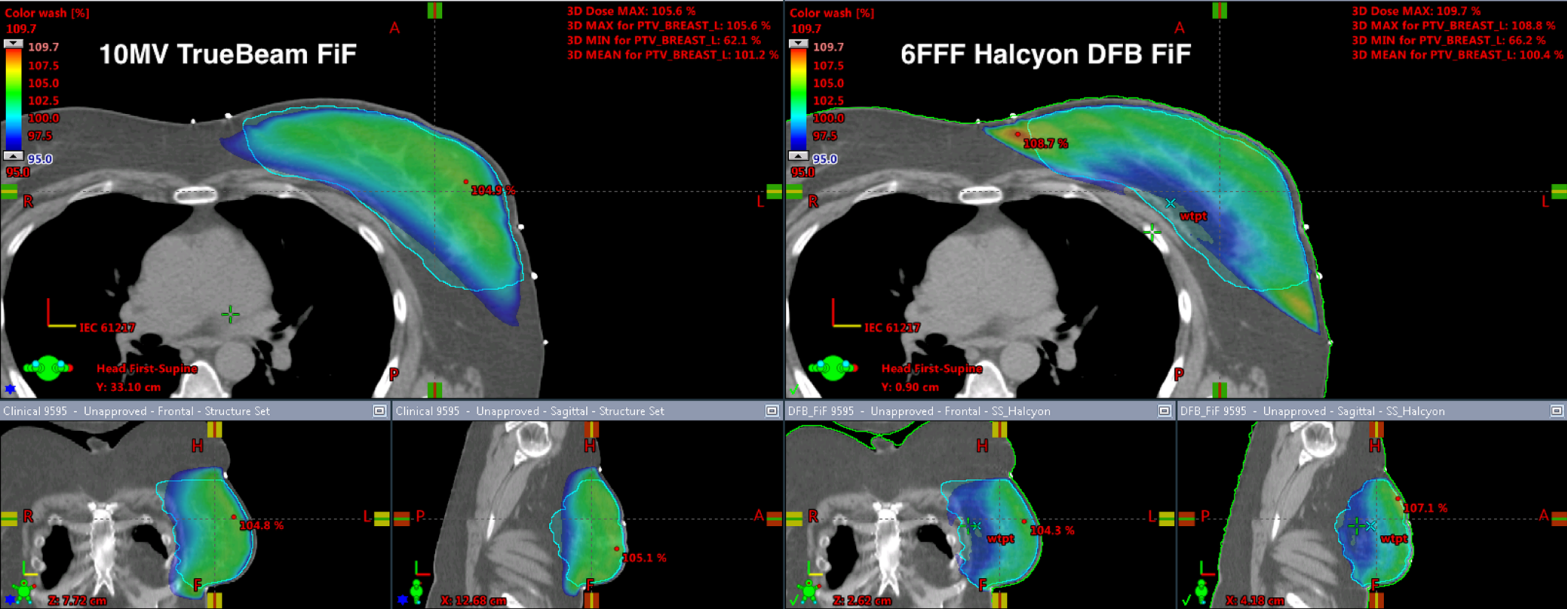
Qualitative analysis for patient 09: Bridge separation = 26 cm. PTV volume = 1129 cc. PTV, planning target volumes.

**Figure 12:**
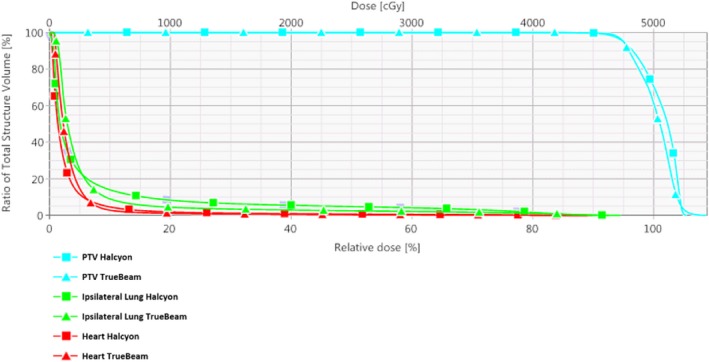
DVH comparison for patient 09: Bridge separation = 26 cm. PTV Volume = 1129 cc. DVH, dose volume histogram; PTV, planning target volumes.

Figures 11 and 12 illustrate the dose distribution and DVH comparisons, respectively, for the most difficult plan encountered across the 10 test patients. This patient had a bridge separation of 26 cm and a PTV volume of 1129 cc which was the largest bridge separation and second largest PTV volume in the study. This patient was clinically treated using only 10MV beams. Qualitatively, the Halcyon does have a less uniform dose but a clinically comparable PTV coverage based on the DVH. The global D_max_ was 3.7% higher (105.6% vs 109.7%). Ipsilateral mean lung dose decreased from 941 cGy to 890 cGy, a 5.4% reduction. The DVH in Figure 12 shows comparable PTV coverage and OAR sparing between the two plans. Overall, the Halcyon plan only incorporated 4 more FiF segments compared to the TrueBeam plan (4 vs 8) (13).

**Figure 13:**
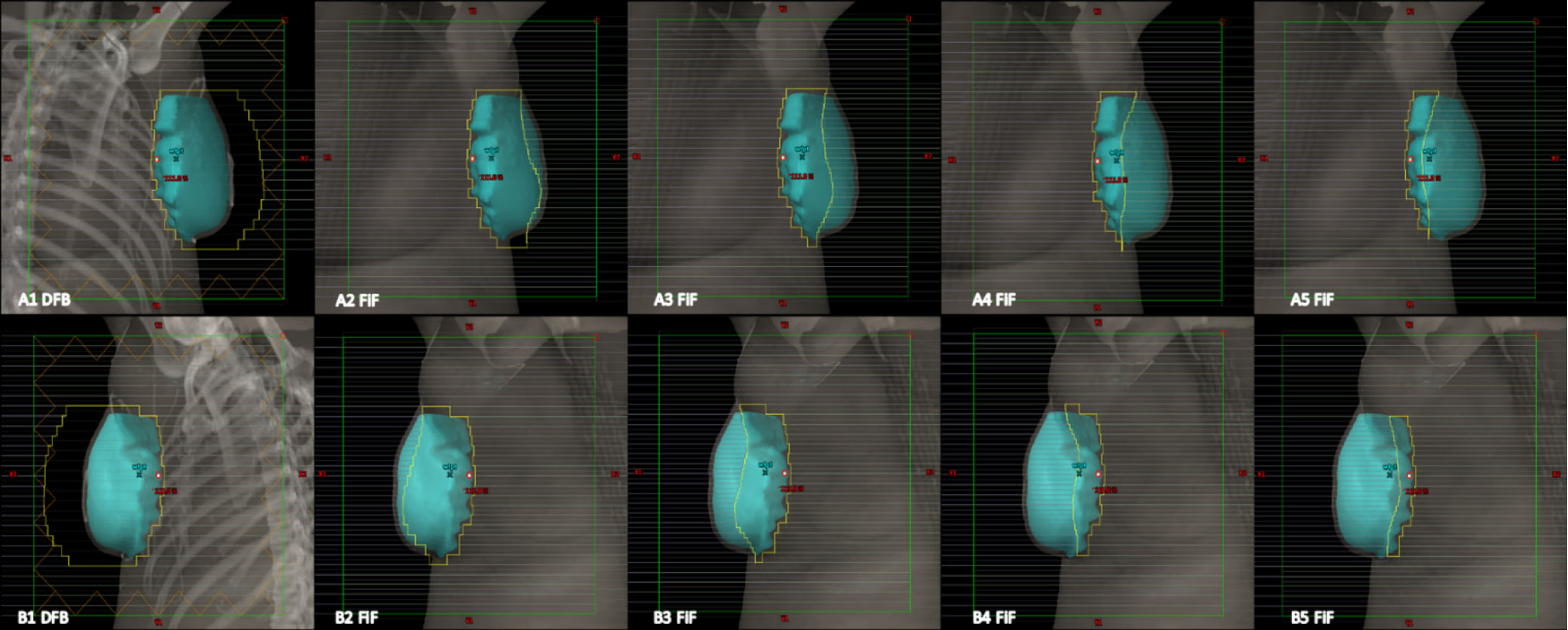
BEV of the DFB and FiF segments for patient 09. Bridge separation = 26 cm. PTV Volume = 1129 cc. BEV, beams‐eye‐view; PTV, planning target volumes.

### 3.3 Deliverability and Plan QA

All FiF plans were successfully delivered on Halcyon. Table 4 shows the ion chamber measurements and gamma passing rates for all ten plans. The average IC measurement had a percent dose deviation of 1.51% with the maximum deviation of 2.23%. All portal dosimetry plans passed 3%/2 mm with 100% points passing. Average Delta4 passing rate was 99.4%.

**Table 4:** Patient‐specific QA results (%).

Patient	IC Point Dose Deviations (%)	PD Gamma Passing Rate (%)	D4 Gamma Passing Rate (%)
1	1.48	100	98.9
2	0.61	100	100
3	0.60	100	99.5
4	1.90	100	100
5	2.06	100	100
6	1.61	100	98.7
7	1.42	100	98.8
8	1.98	100	99.0
9	1.20	100	99.4
10	2.23	100	99.2
Average	1.51	100	99.4

IC = ion chamber, PD = portal dosimetry, D4 = Delta4. Dose difference criteria was 3%, distance to agreement was 2 mm, and 95% gamma passing rate.

## 4 DISCUSSION

We demonstrated a FiF breast planning strategy utilizing a 6FFF beam on the Halcyon platform. All ten plans were determined clinical acceptable. Conventionally, FFF beams have not typically been used in breast 3D planning due to the insufficient ability to deliver homogeneous dose to targets at depth. However, the percent‐depth‐dose (PDD) for both 6 MV flattened (TrueBeam) and unflattened (Halcyon) beams on central axis (CAX) are very similar to one another. The PDD at a 10 cm depth with a 10 × 10 cm^2^ field size and 100 cm source‐to‐surface distance (SSD) for a 6 MV flattened beam is nominally 66.7% vs 63.2% for a 6FFF beam. For comparison, a 10 MV beam has a nominal PDD of 73.2% under the same setup conditions. This similar beam quality gives rise to the hypothesis that with proper beam modulation, the nonflattened profile of a 6FFF beam can be used to yield acceptable target heterogeneity while limiting hot spots of dose outside the targeted breast with a FiF technique. This was even demonstrated for two patients that were clinically treated with 10 MV beams to achieve the deep depth target coverage without exceeding physician defined global D_max_ constraints. The focus of this paper is to establish a planning strategy that makes best use of the technique available to the user of Halcyon linac and to test the feasibility of applying FiF technique to breast patient with jawless design and DSMLC (Double‐Stack MLC). Therefore, we are not expecting to exactly mimic all beam parameters as in clinical plans. The purpose of showing TrueBeam data is not to determine which planning technique or modality is better rather than providing some clinical references.

The primary organs at risk evaluated in this study were the heart, ipsilateral lung, and contralateral lung. The mean heart dose increased by an average of 51.6 cGy using the Halcyon plans (98.0 cGy vs 149.6 cGy). D_max_ to the heart for left sided targets increased by 46.7 cGy (4403.4 cGy vs 4450.1 cGy) and D_max_ decreased for right sided targets by 114.9 cGy (474.9 cGy vs 360.0 cGy). This was due to the initial planning strategy of fitting the primary DFB beams to the PTV with an arbitrary, isotropic 0.3 cm margin. This was chosen to simplify and standardize the planning process for this study. When visualized in the treatment planning system beams‐eye‐view, part of the heart is exposed to the direct beam in the Halcyon plans as a result of this 0.3 cm margin. In clinical practice, the physician would aid the dosimetrist in defining the block edge between the ipsilateral lung, heart and PTV. With the individually designed heart block, the heart dose can be further reduced in the Halcyon plans.

The contralateral lung dose was also higher with the Halcyon plans by an average of 12.6 cGy (39.0 cGy vs 26.4 cGy). The uncertainty in this value, however, is too high to make any deterministic conclusions. It is well‐known that the uncertainty in calculating out of field dose predicted by modern day treatment planning systems is very high,^12^ especially at distances in excess of 10 cm from the field edge. The accuracy of predicting peripheral doses with the Halcyon TPS has also not yet been thoroughly evaluated.

Mean dose to the ipsilateral lung decreased with the Halcyon plans by 15.8% (569.4 cGy vs 676.3 cGy). Similarly to the heart dose, this result could be a function of the isotropic 3 mm margin used in this study. However, the BEV of the clinical TrueBeam vs Halcyon plans is nearly identical when evaluating the block edge relative to the lung/chest wall interface. The softer spectrum of the 6FFF beam is the likely explanation to decreased ipsilateral lung dose. It is evident that the isodoses near the isocenter dip toward the chest wall much more than the TrueBeam 6 MV and 10 MV plans. Lateral scatter and increased attenuation from the medial and lateral lung regions are the most likely causes of a reduction in ipsilateral lung dose when utilizing a 6FFF for breast radiotherapy.

The Halcyon FiF plan requires more MU due to the use of DFB. However, this will not increase the treatment delivery time compared to TrueBeam plan. The ring gantry on Halcyon has the capability to rotate faster at four rotations per minute (RPM) vs 1 RPM for a conventional TrueBeam. The dose rate is also higher (800 MU/min) than that of a 6 MV flattened beam (600 MU/min) on TrueBeam. For the 10 patients tested, the computed delivery time based on average the MU per plan and gantry rotation speed was 62.4 s for Halcyon and 57.9 s for TrueBeam. The MLC movement speed was not accounted for in this study but is not expected to drastically change the previously mentioned delivery times. Setup times for IGRT treatments will also affect total patient treatment times. Portal images typically used for patient alignment pretreatment are not available on the Halcyon. The options for imaging patient setup include: MV 2D/2D (AP and Lateral), MVCBCT, and kVCBCT. Only one option can be chosen during planning and it must be performed daily. Due to the kVCBCT’s lower imaging dose and fast acquisition time (as low as 16.7 s), we anticipate its wide usage. Image acquisition for Halcyon thus is faster than TrueBeam due to the fourfold increase in gantry rotation speed.^6^ In conclusion, treatment delivery times should be comparable between both treatment machines given that patient setup times are significantly longer than beam on times for this type of treatment.

Some limitations to this planning method exist. Firstly, only ten patients’ plans were evaluated, although these ten patients reflect a good laterality and PTV volume distribution, more cases will be investigated in a future study to further characterize the benefits and limitations of breast 3D FiF planning with Halcyon. All Halcyon FiF plans passed the QA indicated the feasibility of the actual delivery of these plans. In our center, we perform hand calculations via a commercial software as our QA approach for current clinical FiF breast plans. On Halcyon, however, since we are using DFB technique, we propose to perform measurement‐based patient‐specific QA, like IMRT cases, as our secondary check. Therefore, there is no direct comparison with clinical plans on measurement‐based QA. As Halcyon is still a relatively new modality and DFB is a new technique, we proposed to use measurement‐based patient‐specific QA for the FiF plans in this study. This does require extra machine time and effort from the physics and QA team compared to traditional FiF plans on TrueBeam machines. It is also important to note that not all breast cancer patients would be eligible for treatment on the Halcyon unit. The maximum field size on halcyon is 28 × 28 cm^2^ at isocenter, therefore patients with a large target size might not fit into the beam portal design. Obese patients may also not be compatible even with the 100 cm diameter physical bore size. From this planning study, for bridge separation distances exceeding 26 cm, it is difficult to achieve adequate coverage at depth with the 6FFF beam quality. In conventional multi‐port 3D breast radiotherapy, additional fields are used to treat involved lymphatics such as the axillary, supraclavicular, and internal mammary lymph nodes. Historically, couch kicks (rotation of the treatment couch relative to the gantry) are used to match field edges to avoid potential under or over‐dosage of normal and targeted tissues. Halcyon is currently not capable of rotating the couch to match beam divergence. One historic alternative to couch rotations is the half‐beam block technique.^3^ This technique utilized the jaws to eliminate beam divergence by placing one of the jaws at the center of the field which will abut subsequent treatment fields. The fact that Halcyon is jawless does not prevent an equivalent approach using the onboard MLC. Leakage is significantly reduced but not eliminated by the Halcyon MLCs compared to the Millenium‐120 which is standard on most TrueBeams. The biggest challenge of Halcyon compared to TrueBeam is the reduced field size at isocenter paired with the increased limitations in isocenter placement due to the physical bore. Future work will include investigation of using Halcyon to treat these complex breast cases.

## 5 CONCLUSION

In this study, a practical and efficient planning method for delivering 3D conformal breast radiotherapy using the Halcyon linear accelerator has been developed. When normalized to the clinically desired coverage, hot spots were maintained to acceptable levels and overall plan quality was comparable to plans delivered on conventional C‐arm LINACs. Using the original isocenter and gantry angles that were used for TrueBeam delivery, the Halcyon patients would not have experienced clearance issues as indicated by the TPS. Intrinsically, the 6FFF beam’s slightly shallower PDD did not prohibit its use for achieving deep target coverage while keeping superficial hot spots below 115% of prescription.

## CONFLICT OF INTEREST

This research study is supported by funding from Varian Medical System. Dr. BC receives grant from Varian Medical System outside submitted work. Dr. SM receives income for consulting from Varian Medical System for work that is outside of this study. Dr. GH reports grants from Varian Medical Systems, grants from ViewRay, Inc., outside the submitted work.
